# Use of an over-the-scope clip in managing adult anastomotic ulcer bleeding^†^

**DOI:** 10.1093/jscr/rjad350

**Published:** 2023-06-30

**Authors:** Anson Y Lee, Scott K Kuwada

**Affiliations:** Department of Medicine, John A. Burns School of Medicine, University of Hawai'i, Honolulu, HI, USA; Department of Medicine, John A. Burns School of Medicine, University of Hawai'i, Honolulu, HI, USA; The Queen’s Medical Center, Honolulu, HI, USA

## Abstract

Bleeding from anastomotic ulcers following surgical procedures such as ileocolonic resection in patients without Crohn’s disease is a rare occurrence and difficult to manage. Although a number of treatment options have been explored, they have all had varying success. This case characterizes the first reported successful treatment of recurrent gastrointestinal bleeding in an adult due to an anastomotic ulcer with an over-the-scope clip.

## INTRODUCTION

Anastomotic ulcers (AUs) following surgical procedures such as ileocolonic resection in patients without Crohn disease are a rare occurrence and are often only characterized in case reports and series [1]. Although primarily present in the pediatric population [2, 3], the overall incidence of AUs among both adult and pediatric patients has been estimated to be between 0.3 and 8% [4–7]. After AUs were identified on the first postoperative colonoscopy, they were found to persist in 80% of patients regardless of various medical therapies [1, 8]. The primary symptoms of AUs include abdominal pain, iron deficiency anemia and evidence of gross or occult gastrointestinal (GI) bleeding [1, 2, 6]. Potential causes of AUs include the use of non-steroidal anti-inflammatory drugs (NSAIDs), development or recurrence of Crohn Disease or malignancy, toxic effects of unabsorbed bile, suture material and ischemia [9–11]. However, effective treatment options for AU are lacking, and physicians often resort to resecting the affected AU with re-anastomosis of the ileocolonic region [1]. To date, no study has reported the use of an over-the-scope clip (OTSC) in the successful closure of an AU in an adult patient to treat recurrent bleeding.

## CASE REPORT

The patient is a 67-year-old woman with a complex medical history of pulmonary fibrosis, rheumatic heart disease, status post mitral commissurotomy and mechanical mitral valve replacement; with warfarin, atrial fibrillation, benign colon mass, status post colostomy, and history of two recurrent episodes of ileocolonic anastomosis bleeding. She presented to the emergency department with progressively bloody small volume ‘plum-colored’ stools over 3 days but continued to take warfarin. Family history was relevant for stomach cancer in her father but negative for any other GI diseases or bleeding disorders. Physical exam was negative for abdominal tenderness but did identify an irregular, systolic ejection murmur at the left sternal border. Laboratory studies conducted on her day of presentation identified a glucose of 122 mg/dl, red blood cell count of 3.21 million/L, hemoglobin of 9.1 g/dl, hematocrit of 28.2%, prothrombin time of 29.2 s, partial thromboplastin time of 37.4 s and international normalized ratio of 2.9. The comprehensive metabolic panel as well as all other complete blood count values were normal. A same-day computed tomography angiogram for acute gastrointestinal bleeding revealed a lack of active GI tract bleeding. She had no antibiotic exposures or changes in diet and was admitted for an in-patient stay.

**Figure 1:**
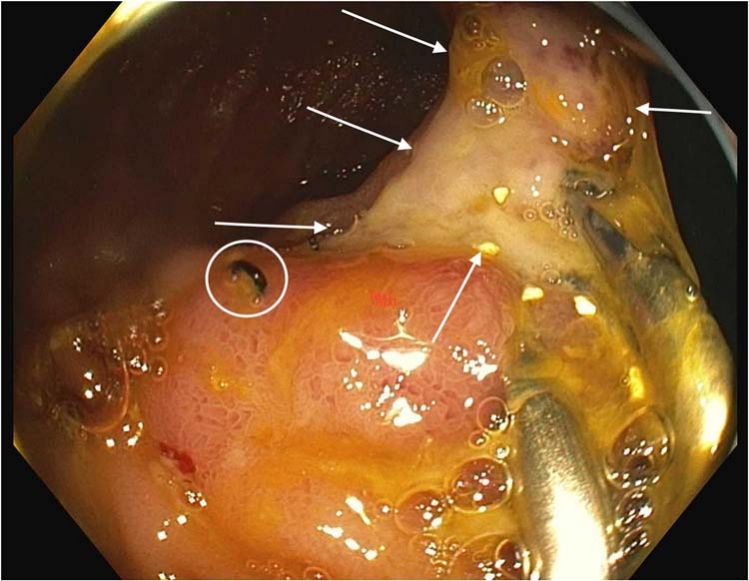
Anastomotic ulcer prior to OTSC use. Arrows show anastomotic ulcer. Circle shows surgical staple at anastomosis. Hemostatic clips that were previously applied to attempt hemostasis are present on the right side of the ulcer.

On Day 2, both an endoscopy and colonoscopy were performed on the patient. Endoscopy exhibited a small hiatal hernia without Cameron's erosions and a solitary tiny gastroesophageal junction polyp. The duodenum was normal, and no specimens were collected or polyps were biopsied due to the patient’s active bleeding. Initial colonoscopy impressions identified a normal terminal ileum, blood in the examined colon, as well as erythema, friable mucosa and ulceration on the patient’s functional end-to-side ileocolonic anastomosis. A solitary ulcer was identified at the colonic anastomosis which was actively secreting blood. Hemoclips were placed for hemostasis (Fig. 1).

**Figure 2:**
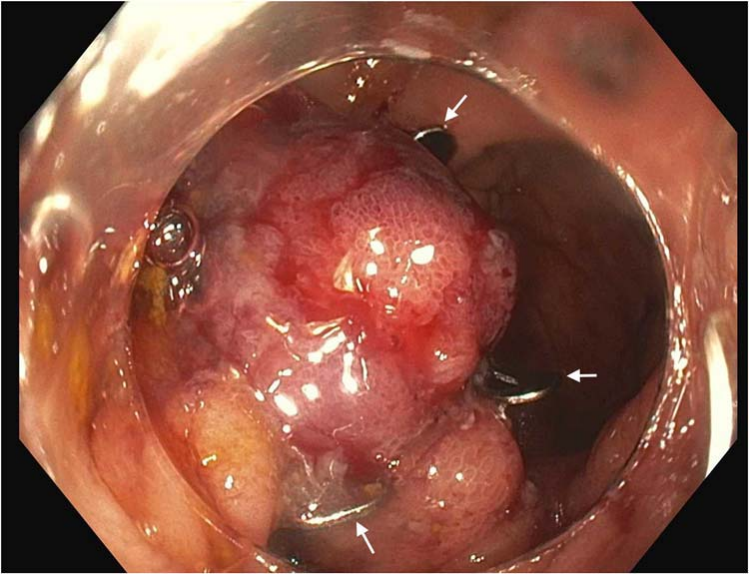
Anastomotic ulcer following OTSC use. Arrows show edges of the OTSC that was deployed on the ulcer. Approximated margins of the ulcer are visible within the clip.

Eight days later, a repeat colonoscopy was conducted due to recurrent hematochezia and acute posthemorrhagic anemia. A prior end-to-side ileocolonic anastomosis was visualized at the transverse colon. The distal ileum demonstrated a solitary ulcer, which was 7 mm in diameter at the ileocolonic anastomosis. The ulcer was surrounded by four hemoclips that were subsequently removed with rat-toothed forceps to provide access to the ulcer edges prior to clip removal. Minor bleeding was present at the edges of the ulcer. Following these observations, a single Ovesco 1416 t clip (Ovesco Endoscopy AG, Tübingen, Germany) was loaded onto the colonoscope and advanced to the ileal ulcer. Tissue edges were approximated with a twin grasper device, and suction applied to draw the ulcer into the cap device. A single 14 mm OTSC was then positioned and delivered across the entirety of the ulcer (Fig. 2). Closure of the AU was successful, and hemostasis achieved. The patient was then returned to the hospital ward and told to continue prescribed medications as well as a clear liquid diet. On Day 16 post-admission, the patient was discharged home. Since the OTSC placement, the patient has not had recurrent lower GI bleeding or worsening anemia for over 9 months, while continuing on chronic warfarin.

## DISCUSSION

AUs are often difficult to manage, and in this case, the patient presented with overt GI bleeding and anemia. Previous studies have outlined a number of potential management options for AUs, but have had variable success. In all patients, it was found that NSAIDs should be ceased immediately, and any inflammatory bowel disease should be treated [1]. In terms of therapeutic options, sulfasalazine, sucralfate, misoprostol, 5-aminosalicylic acid and antacid therapy have all been utilized but with varying results [1–3, 6, 7].

However, endoscopic treatments for AUs carry a lot of promise. Barraclough *et al*. presented a series of pediatric patients who underwent different treatment options for AUs [9]. They concluded that prompt treatment should be started once AUs were detected, ideally using endoscopic techniques which included a combination of endoclipping and argon photocoagulation, especially if patients did not require blood transfusions [9]. Another endoscopic treatment option is the use of OTSCs. OTSCs are increasingly being employed as a non-surgical modality for treating adult GI hemorrhages, ulcer bleeding, perforations, fistulas and anastomotic leaks [12–14]. Although not created intentionally for the closure of AUs, it proved to be beneficial in the current patient. She required only a single OTSC after hemoclips failed to achieve hemostasis.

This case characterizes the first reported successful adult AU closure with an OTSC. The novel use of such a device for AUs emphasizes the importance of experiences described in case reports. This practical and simple application of an OTSC opens up the possibilities of its use in future practice.

## Data Availability

Data sharing is not applicable to this article as no new data were created or analyzed in this study.

## References

[1] Chari ST, Keate RF. Ileocolonic anastomotic ulcers: a case series and review of the literature. Am J Gastroenterol 2000;95:1239–43. 10811334 10.1111/j.1572-0241.2000.02016.x

[2] Sondheimer JM, Sokol RJ, Narkewicz MR, Tyson RW. Anastomotic ulceration: a late complication of ileocolonic anastomosis. J Pediat J 1995;127:225–30. 10.1016/s0022-3476(95)70299-7 7636646

[3] Hamilton AH, Beck JM, Wilson GM, Heggarty HJ, Puntis JW. Severe anaemia and ileocolic anastomotic ulceration. Arch Dis Child 1992;67:1385–6. 1471894 10.1136/adc.67.11.1385 PMC1793781

[4] Fusaro F, Tambucci R, Romeo E, Bagolan P, Dall'Oglio L, Ceccarelli S, et al. Anastomotic ulcers in short bowel syndrome: new suggestions from a multidisciplinary approach. J Pediatr Surg 2018;53:483–8. 28610705 10.1016/j.jpedsurg.2017.05.030

[5] Charbit-Henrion F, Chardot C, Ruemmele F, Talbotec C, Morali A, Goulet O, et al. Anastomotic ulcerations after intestinal resection in infancy. J Pediatr Gastroenterol Nutr 2014;59:531–6. 24979478 10.1097/MPG.0000000000000472

[6] Weinstock LB, Shatz BA. Endoscopic abnormalities of the anastomosis following resection of colonic neoplasm. Gastrointest Endosc 1994;40:558–61. 7988818 10.1016/s0016-5107(94)70252-7

[7] Peter Z, Bodoky G, Szabo Z, Sonfalvi E, Varga Z, Szilvasi I. Ileocolic anastomotic ulcer after surgery in adulthood: case report and review of the literature. Z Gastroenterol 2004;42:605–8. 15248109 10.1055/s-2004-813231

[8] Hirten RP, Ungaro RC, Castaneda D, Lopatin S, Sands BE, Colombel JF, et al. Anastomotic ulcers after ileocolic resection for Crohn's disease are common and predict recurrence. Inflamm Bowel Dis 2020;26:1050–8. 31639193 10.1093/ibd/izz224 PMC7456972

[9] Barraclough H, Girach A, Rao P, Urs A, Marven S, Murthi G, et al. Anastomotic ulcers: a tertiary Centre experience of endoscopic management techniques. J Pediatr Gastroenterol Nutr 2021;73:329–32. 33938524 10.1097/MPG.0000000000003159

[10] Bhargava SA, Putnam PE, Kocoshis SA. Gastrointestinal bleeding due to delayed perianastomotic ulcers in children. Am J Gastroenterol 1995;90:807–9. 7733091

[11] Pateria P, Chong A. A recurrent, ischaemic ileocolonic anastomosis ulcer refractory to surgery treated with hyperbaric oxygen. Diving Hyperb Med 2018;48:194–6. 30199892 10.28920/dhm48.3.194-196 PMC6205864

[12] Bartell N, Bittner K, Kaul V, Kothari TH, Kothari S. Clinical efficacy of the over-the-scope clip device: a systematic review. World J Gastroenterol 2020;26:3495–516. 32655272 10.3748/wjg.v26.i24.3495 PMC7327783

[13] Xiao X, Lau JY. Over-the-scope clip treatment of refractory peptic ulcer bleeding. Gastrointest Endosc 2016;83:458–9. 26773640 10.1016/j.gie.2015.05.040

[14] Masaki S, Yamada K. Over-the-scope clip closure of persistent gastrocutaneous fistula after percutaneous endoscopic gastrostomy tube removal: a report of two cases. Cureus 2021;13:e13206. 33728166 10.7759/cureus.13206 PMC7946610

